# iPad-Assisted Measurements of Duration Estimation in Psychiatric Patients and Healthy Control Subjects

**DOI:** 10.1371/journal.pone.0061295

**Published:** 2013-05-02

**Authors:** Irene Preuschoff, Helge H. Müller, Wolfgang Sperling, Teresa Biermann, Matthias Bergner, Johannes Kornhuber, Teja W. Groemer

**Affiliations:** Department of Psychiatry and Psychotherapy, Friedrich-Alexander-University of Erlangen Nürnberg, Erlangen, Germany; Duke University, United States of America

## Abstract

Handheld devices with touchscreen controls have become widespread in the general population. In this study, we examined the duration estimates (explicit timing) made by patients in a major general hospital and healthy control subjects using a custom iPad application. We methodically assessed duration estimates using this novel device. We found that both psychiatric and non-psychiatric patients significantly overestimated time periods compared with healthy control subjects, who estimated elapsed time very precisely. The use of touchscreen-based methodologies can provide valuable information about patients.

## Introduction

Mobile computing has become very popular in recent years. Handheld devices with touchscreens, tablets and especially smartphones, are now commonly used by the general population and as instruments for data collection also used in clinical and academic settings [1], and their validity and practicality have been demonstrated in a wide range of settings and for a variety of users [2], [3]. However, usability studies indicate that data collection with small handheld devices (e.g., PDAs) yields less accurate data because users struggle with small screens or onscreen keyboards [1]. Haller et al. (2009) conclude that researchers could overcome these methodological difficulties by using tablet PCs, which combine the advantages of laptops and PDAs. To date, no studies have evaluated touchscreen tablet PCs for psychiatric testing. Thus, the aim of the present study was to exemplary demonstrate data collection using a touchscreen tablet for psychiatric patients performing a repeated task in a clinical trial. In the present study, we thus introduce a new application on a touchscreen handheld device as a tool for time estimation tasks.

Time experience [4] is a repeatedly investigated phenomenon in psychological and psychiatric research [5]–[8], and a “remarkable range that accrues across different individuals” [9] and in groups of psychiatric diseases like substance misuse or iatrogenic treatments [10]–[12] has been revealed. In studies concerning this issue, many aspects of time experience, such as time production or time estimation (see table 1 for an overview of terminology), have been investigated using various methods, such as verbal estimation tasks [8] or computerized assessment tools [11], [13] or as part of assessment batteries [14].

**Table 1:** Terminology in time sense research (modified from Bschor et al., 2004).

term	explanation
time sense/time experience	all aspects associated with the experience of time flow
time awareness/time perception/subjective speed of time	the subjective experience of how fast or slow time is passing
time judgment	a subject's objectively measured capacity to judge the length of a given timespan
time estimate/duration estimate/explicit timing	verbal judgment of the subjective length of a given timespan
time production	production of a certain timespan, e.g., by saying “start” and “stop”
time underestimation	timespans are estimated as shorter than they are, or produced time periods are longer than requested
time overestimation	timespans are estimated as longer than they are, or produced time periods are shorter than requested

Several studies have been conducted to compare the time sense of psychiatric patients with that of healthy control subjects [8], [12], [15]–[18] with different assessment paradigms. Tysk (1984) observed time underestimation in patients suffering from depressive states, whereas manic patients tended to overestimate time intervals. On the other hand, in a time reproduction task of 1, 6, and 37 s it was observed that manic patients underreproduced time intervals, whereas patients with major depression overreproduced these timespans [18]. Another study showed an overestimation of 8-, 49-, and 109-s time intervals in both depressed and manic patients [17]. In a study of time estimation tasks comparing patients suffering from major depression with healthy subjects the patients with depression overestimated intervals longer than 10 s but estimated shorter timespans correctly [19], whereas no statistically significant difference between the groups was found in another recent study [8].

Further, patients with schizophrenic disorders and patients with depression and dysthymic disorders with healthy control subjects were compared with regard to their ability to discriminate time intervals in the range of milliseconds. Whereas schizophrenic patients and patients with affective disorders exhibited “highly reduced performance in temporal discrimination” the healthy control subjects produced fairly accurate results [5].

Research concerning time sense has also been conducted in individuals with psychoactive substance abuse disorders [11], [12], [20], [21], mild cognitive impairment and Alzheimer's disease [22], [23].

In summary, most of these studies have revealed that time perception is impaired in psychiatric patients, especially in patients with major depression and schizophrenia, but in certain cases, the findings are inconsistent. These inconsistencies could be due to limitations such as small sample sizes or unsuitable time intervals. Moreover, the use of a broad range of tasks labeled “time experience” or “time estimation” with various assessment paradigms makes it difficult to accurately compare different datasets.

Few studies have evaluated the suitability of time interval lengths in time estimation tasks. Tasks using intervals in the range of milliseconds investigate time perception that is less influenced by cognitive processes than tasks that present the subjects with time intervals in the range of seconds or minutes. In the former tasks, cognitive processes, such as attention and memory, mediate time estimation [5], [19] postulate that 3 s is a suitable time unit for time estimation and can serve as an “internal benchmark”, but there is a lack of empirical evidence for this supposition.

In the current paper, we introduce a touchscreen-based task for duration estimation tasks and provide methodological time interval metrics that can be used. We found that in a sample of the general population, duration estimation was very accurate and precise, whereas psychiatric inpatients overestimated durations. We conclude that meaningful data can be acquired with interactive touchscreen tasks in various environments.

## Methods

### Task

**Figure 1:**
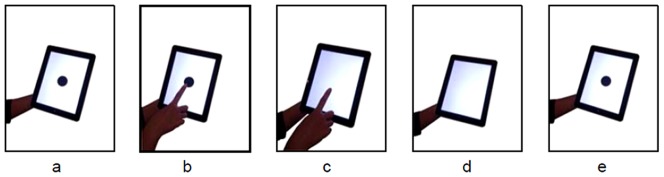
Time estimation task. A) To begin, a black symbol was shown on the screen. B) and C) When the participant touched the screen, the symbol vanished. D) The participant was asked to look at the screen. E) When the stimulus appeared again, the subjects had to estimate the duration of the time that had passed.

Duration estimation was assessed with the following interactive task (Figure 1). A black symbol was displayed in the center of the touchscreen when the application began. In most of the trials, the symbol was a circle. To avoid symbol bias, a square (instead of a circle) was presented as a control stimulus. When the participant touched the screen, the symbol vanished and flashed after a certain time period. When the symbol re-appeared, the subjects had to estimate the duration of the interval. There was no fixed interval between the trials; the subjects had to tap the screen to proceed to the next trial. After three trials, the task was finished.

The subjects were given a standardized verbal review of the procedures. If necessary, the standardized briefing was repeated during the task or short explanations were given. Feedback was provided upon request after the three tasks were completed.

A subgroup of 135 individuals (91 psychiatric patients, 21 healthy control subjects, and 22 patients with somatic symptoms) was questioned about their subjective time sense after the standardized time estimation task.

### Calculation of duration estimation errors

The relative error was calculated as
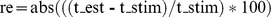
with re = relative error, t_est = estimated duration [s], t_stim = length of time before the cursor vanished [s]

### Participants

#### Psychiatric subjects

The study included 149 adults (87 females) from three open and two closed psychiatric wards and a day-care ward. To allow for the use of a handheld touchscreen device in the clinical trial, patients who were awake, accessible, and responsive were included. The subjects were approached in their acute environment (mostly rooms or day rooms) and asked to participate in a time estimation task. To avoid disturbing the daily routine of the wards, there was no fixed time of day for data collection. The patients were asked to participate when they were accessible and had spare time. Before data collection, the nursing staff members were asked to identify the patients who did not have the capacity to consent (e.g., because of severe dementia). These patients were not included in the study.

After each patient's diagnosis was recorded, the daily medications and number of days in the hospital according to the medical record were registered. Psychotropic medications were defined as substances belonging to the following groups: antidepressants, antipsychotics, tranquilizers, mood stabilizers, and stimulants. Somatic patients that took medications from one of these groups were excluded.

All of the patients had signed a general agreement that psychometric data could be collected during their stay in the hospital and used anonymously for scientific studies. Thus, no additional written consent from the patients was necessary.

Diseases were classified according to the ICD-10 (International Statistical Classification of Diseases and Related Health Problems, 10th revision, as published by the World Health Organization). The following classifications from the chapter on “mental and behavioral disorders” (Chapter “F”) were used:

F0: Organic, including symptomatic mental disorders, e.g., dementia;

F1: Mental and behavioral disorders due to psychoactive substance abuse, e.g., mental and behavioral disorders due to alcohol abuse;

F2: Schizophrenia schizotypal and delusional disorders;

F3: Mood [affective] disorders, e.g., recurrent depressive disorder;

F4: Neurotic, stress-related, and somatoform disorders, e.g., panic disorder [episodic paroxysmal anxiety];

F5: Behavioral syndromes associated with physiological disturbances and physical factors, e.g., anorexia nervosa;

F6: Disorders of adult personality and behavior, e.g., emotionally unstable personality disorder;

F7: Mental retardation;

F8: Disorders of psychological development;

F9: Behavioral and emotional disorders with onset usually occurring in childhood and adolescence

### Controls

Two control groups were recruited. One group consisted of 111 healthy controls (59 females) who participated in the time estimation task. To create an environment that was fairly comparable to the patients' environment (generally, their rooms), the healthy subjects were not approached in very noisy surroundings but rather while sitting on a park bench, in a café, or in a similar setting. Students, hospital staff, and the authors' friends were solicited for inclusion in the healthy control group. They confirmed not to take psychotropics or suffer from mental illness. Twenty-two patients (12 females) who attended different clinics (e.g., general surgery, surgery due to accidents, and internal medicine) and who were not using psychotropic medication were also included in the study. The subjects were not reimbursed for their participation. In both control groups, age and gender were the only personal data collected. For this reason, following the guidance of an ethics consultant, no additional written consent was necessary.

### Stimuli and Procedure

Intervals from 1 to 60 s were randomly generated using custom MatLab (The Mathworks, USA) routines before starting data collection every day, and the parameters were set using the parameter screen of the application.

### Hardware

Measurements were recorded using a common, commercially available, first-generation Apple iPad (Apple Inc., 2010).

### Application for the time estimation task

The application was written in Cocoa Touch by a professional programmer (mani.de). The start screen offers the choice of either performing a test or setting the parameters. The possible parameters were as follows:

Cursor size (diameter or perimeter, cm)

Cursor shape (circle, square, or triangle)

Number of trials (n)

Duration of the cursor's appearance (s)

Color of the cursor (RGB)

Color of the background (RGB)

For most measurements, we performed 3 trials with a round black cursor, 3 cm in diameter, on a white background. For some of the measurements, the cursor was a square.

After performing the specified number of trials, the screen was set to display a message (“end of the test”), which vanished when the participant touched the screen, and the screen returned to the start screen.

### Methodical considerations and evaluation

We wanted to generate a duration estimation task that was controlled by the patient. We chose a self-controlled visual cue for two reasons: 1) to avoid surprise and 2) because the acoustic environment was not well controlled in the different wards and in the settings where we met the healthy control subjects. However, these are typical settings for assessments using mobile devices with touchscreens. According to Vispoel et al. [24], examinees who are taking computerized tests “desire as much control (…) during the test as they can possibly have” (p 75). With this idea in mind, as well as to minimize investigator bias, we developed a task that requires the patient to interact with the device to start the measurement. As mentioned above, there is no empirical evidence or general paradigm that defines timespans for time estimation tasks. Thus, we generated timespans based on the following logical assumptions: 1) the time unit should be known and familiar to all subjects (which excluded milliseconds); 2) measurements must be performed in a limited amount of time to obtain large sample sizes; and 3) answers with different time units should be avoided for good comparability. Given these considerations, the use of milliseconds seemed unsuitable because most subjects would not be familiar with describing time experiences using this unit. We decided to use seconds as the estimation unit for timespans up to 60 s because the aim was to estimate the interval in seconds and to avoid imprecise answers. We assumed that intervals that can be measured in seconds would be estimated in seconds and that intervals that can be measured in minutes would be estimated in minutes. This assumption is based on common sense, although we did not find a supporting reference after conducting a thorough literature search and consulting with experts. Three time intervals ranging from 1 to 60 s were randomly generated before collecting data every day. The statistical analysis showed that the randomly generated intervals were equally distributed (Z = .79, p = .57, n.s.).

### Analysis software

The data were analyzed with SPSS 18 (Statistical Package for the Social Sciences) or MatLab(c).

## Results

### Abbreviations and nomenclature

In the following section, we use “*M*” to denote medians and “*SD*” to denote standard deviations. Thereby the variable of concern is denoted in parenthesis. *M(re)* thus would mean “median of the relative error”. The subgroup values are denoted as “value-abbreviation_subgroup-feature1_subgroup-feature2” (e.g., “*M(re)*_male_patients” denotes the median value for the relative error of male patients).

### Overall statistics

During the 2-month assessment period, a total of 338 subjects were asked to perform the time estimation task with the iPad, and more than 83% agreed to participate. A total of 282 subjects (158 females) with a mean age of 41.3 years (*SD* 15.2) accomplished the time estimation task. See table 2 for an overview of participants and refusals in the different groups:

**Table 2:** Descriptive statistics of participants and refusals in psychiatric patients, healthy and somatic ill controls.

	participants	mean age	females	refusal	mean age	females	*total*
psychiatric patients	149	44.6 (*SD* 14.6)	87	37	49 (*SD* 16.6)	12	273
healthy controls	111	35 (*SD*13)	59	13	39.9 (*SD* 16.5)	5	124
somatic ill patients	22	51.3 (*SD* 17.1)	12	6	51 (SD 7.7)	4	28
							338

### Descriptive statistics for the psychiatric patients

A total of 186 psychiatric patients (112 females) were asked to participate in the study. The refusal rate was 20%; 37 subjects (25 females) declined to participate or were unable to participate because of their mental status. Thus, 149 (87 females) patients participated in the time estimation task. A chi-square test did not reveal gender-related differences in the refusal rates (*Chi^2^* (2, *N* = 186) = 1.03, n.s.). A t-test demonstrated that the subjects who declined to participate in the task were significantly older than the patients who participated in the study (*t*(183) = 2.59, *p* = .01). The average duration of hospital stay was 17 days (*SD* 18); one-third of the patients were approached within the first four days of their stay. Refusal to participate in the study was not associated with the duration of stay (*t*(180) = 1.00, *p* = .32, n.s.).

The most frequent diagnoses were affective disorders (*n* = 77), followed by neurotic, stress-related, and somatoform disorders (*n* = 26) and schizophrenia and schizotypal disorders (*n* = 21). The other primary diagnoses were dementia (*n* = 6), disorders associated with psychoactive substance abuse (*n* = 6), eating disorders (*n* = 8), personality disorders (*n* = 2), and hyperkinetic disorders (*n* = 1). Three patients had not been given a diagnosis at the time of the assessment. Refusal to participate in the study was not associated with the primary diagnosis (*Chi^2^* (5, *N* = 183) = 10.89, n.s.).

### Descriptive statistics for the healthy control subjects

We invited 124 healthy controls (64 females) to participate in the study; 13 subjects (5 females) declined to participate, yielding a refusal rate of 9%. A total of 111 participants (59 females) completed the time estimation task. Again, a chi-square test did not reveal gender-related differences in the refusal rates (*Chi^2^* (2, *N* = 111) = 1.01, n.s.). In contrast to the psychiatric patients, refusal among the healthy subjects was not associated with age (*t*(121) = 1.21, *p* = .23, n.s.).

A t-test showed that the psychiatric patients were significantly older than the healthy subjects (*t*(249.4) = 5.55, *p* = .000). To prevent a possible age bias, we excluded the data from psychiatric patients older than 53 years. Data from 103 psychiatric patients with an average age of 37.7 years (*SD* = 11.82; *t*(212) = .97, n.s.) were included in further analyses.

### Overall time estimation

For each time estimate, the deviation from the actual value was calculated as a percentage. Next, we calculated the average percent deviation across the three time estimates. First, we were interested in whether we could reproduce the previously reported findings regarding time estimation errors. The distribution of time estimation errors in the control group did not differ significantly from normal (Kolmogorow-Smirnov-test) and thus matched the prerequisite for the Student's t-test. The average estimation error for all subjects was 12.8% (*SD* 51.2). The average estimation error was 23.47% (*SD* 62.53%) for the psychiatric patients and 0.97% (*SD* 32.29%) for the healthy control subjects. T-tests revealed that the differences in the absolute relative errors were highly significant; specifically, the psychiatric patients misestimated timespans, whereas the healthy control subjects produced rather accurate time estimates (*t*(152) = 3.28, *p* = .001). The constant error for all subjects, which represents the deviation of each estimate from the target time, was 2.27 s (*SD* 16.37 s). The constant error, was 6.33 s (*SD* 22.66 s) among the psychiatric patients and −1.73 s (*SD* 7.06 s) among the healthy control subjects. T-tests revealed that the differences in constant error were highly significant; specifically, the psychiatric patients overestimated the timespans, whereas the healthy control subjects produced rather accurate time estimates (*t*(106) = 3.23, *p* = .002).

### Clipping to multiples of 5 s

**Figure 2:**
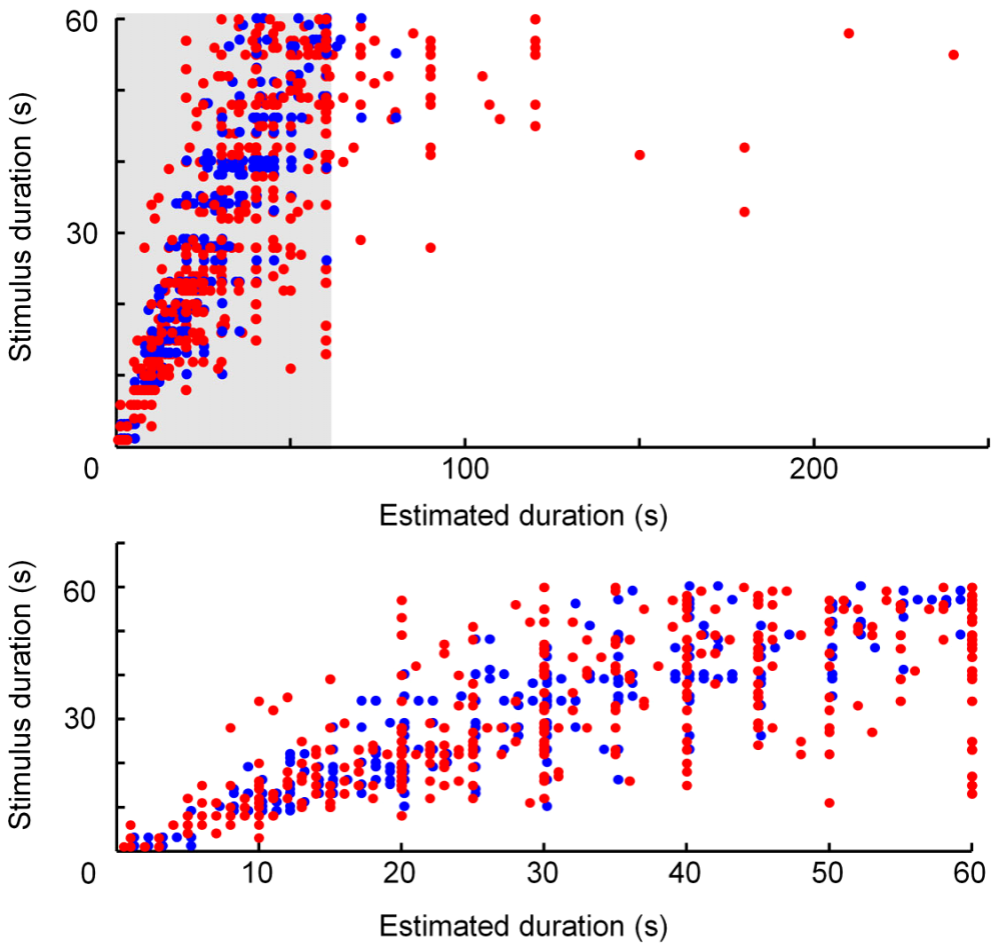
Relationship between stimulus duration and duration estimated by the subjects (blue: control subjects; red: psychiatric patients). Please note that clipping to 5-s and 10-s time intervals is observed in both the control group and the patient group.

Clipping to values that were multiples of 5 s was a phenomenon in the experiments (Figure 2). We however suggest that it is not likely that the statistics were affected by clipping for the following three reasons.

**Figure 3:**
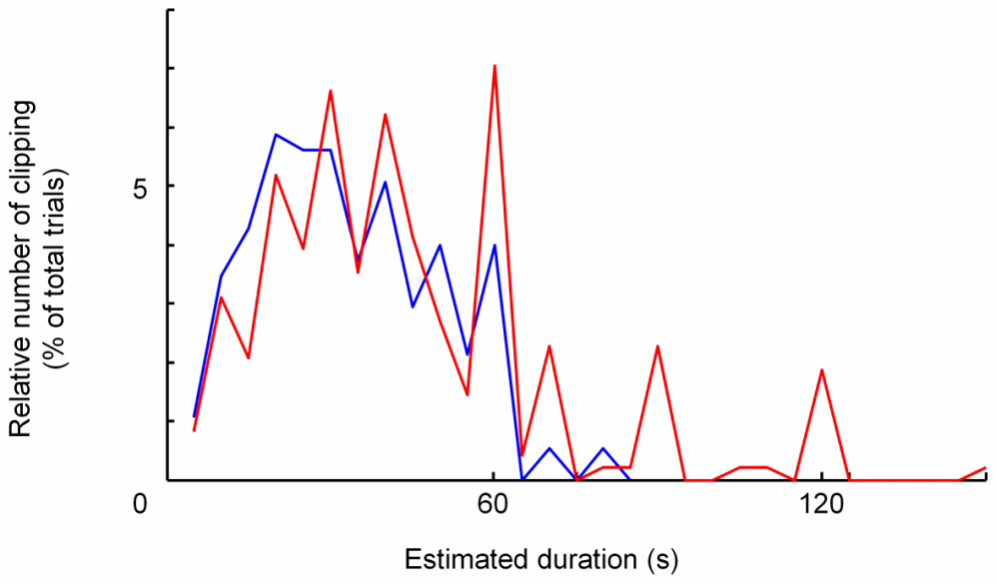
Relative number of clipped values at given times (multiples of 5) (blue: control subjects; red: psychiatric patients). Clipping had a characteristic distribution and was similar in both groups.

1. Clipping is observed in both groups (Figure 2)
2. Clipping bias was reduced by repetitive measurements that lead to average values. These were calculated as the mean of three different estimates produced by one subject in our study.
3. Clipping to multiples of five was observed in the control subjects and the patients. The relative amount of clipping and the clipping distribution were very similar in both groups (Figure 3).

### Suitability of different time interval lengths

Because of the lack of scientific evidence concerning the suitability of various timespans for time estimation tasks, we analyzed estimation errors in 5 s time units. The aim was to determine whether the subjects' estimation errors differed depending on the duration of the time interval. Differences would imply that certain timespans are more suitable for time estimation tasks than others. Thus, the estimation errors for the various timespans defined by 5 * (i+1) : 5 * (i+5); (i = 1∶11) (i.e., 1–5 s up to 56–60 s) were compared using an ANOVA.

For the estimates produced by both the patients and the control subjects, the estimation error was significantly higher for the interval of 1–5 s (*M_*patients = 56.48%, *SD* = 94.0; *M_*controls = 53.70%, *SD* = 104.87) than for the other time intervals. Among the psychiatric patients, these differences failed to reach the level of significance (*F*(11, 432) = 1.47, *p* = .14, n.s.) found in the ANOVA, but among the healthy control subjects, the ANOVA with Scheffé's post hoc test showed that the estimation error for the 1–5 s interval was significantly higher than the error for the other intervals (*F* (11, 320) = 4.40, *p* = .000). However, Scheffé's post hoc tests showed no differences in the estimation errors when we compared them with the errors associated with other time intervals. In the ANOVA with Scheffé's post hoc tests that excluded the data from the 1–5-s interval, there were no differences in the estimation errors for the given intervals (*F_*patients (10, 415) = 1.11, *p* = .35, n.s; *F_*controls (10, 303) = 1.64, *p* = .09, n.s.). We concluded that the data from the 1–5 s interval biased the average estimation errors of the patients and the control subjects. Thus, these data were excluded in further analyses.

The newly calculated relative errors were 20.2% (*SD* 61.15) for the psychiatric patients and −1.18% (*SD* 26.44) for the healthy subjects (*t*(138.28) = 3.29, *p* = .001). All following measurements are given as relative errors with excluded 1–5 s time intervals.

### Effects of repetitive measurements

The time estimation task included three trials per subject. To determine whether estimation error is affected by the repetition of measurements, paired t-tests were conducted. The psychiatric patients produced significantly lower error percentages in the third trial (*M*_patients_third_trial = 11.43%, *SD* = 60.99) compared with the first trial (*M*_patients_first_trial = 28%, *SD* = 63.86; *t*(97) = 3.53, *p = *.001). A comparison between the third and second trials (*M*_patients_second trial = 22.51%, *SD* = 79.32) also showed that the relative error was lower in the third trial, but the difference was not significant (*t*(90) = 1.68, *p = .*10, n.s.). However, there was no significant improvement in error in the second trial compared with the first trial (*t*(92) = 1.25, *p = .*21, n.s.). The healthy control subjects were precise in their estimates from the first trial, and the comparisons did not reveal significant differences between the three trials (see table 3 and 4).

**Table 3:** Paired t-test of repetitive measurements in the group of psychiatric patients.

trial order	*M* (*SD*)		*T* (*df*)	*p*-value
1	28.0 (63.86)	error1–error2	1.25 (92)	.21
2	22.51 (79.32)	error1–error3	3.53 (97)	.00
3	11.43 (60.99)	error2–error3	1.68 (90)	.10

**Table 4:** Paired t-test of repetitive measurements in the control group.

trial order	*M* (*SD*)		*T* (*df*)	*p*-value
1	−1.32 (26.51)	error1–error2	1.75 (91)	.08
2	−3.71 (26.45)	error1–error3	.19 (98)	.85
3	−1.13 (33.84)	error2–error3	−.05 (103)	.96

### Specific characteristics of duration estimates

Further analysis failed to reveal significant gender differences in the time estimates; Overall, women demonstrated a higher relative error in time estimates, but the differences failed to reach significance (*M(re)*_female_patients = 29.05, *SD* = 64.99; *M(re)*_male_patients = 7.12, *SD* = 53.07; *t*(102) = 1.81, *p* = .07, *n.s.*). A gender-related discrepancy was also not observed in the healthy control group (*M(re)*_female_controls = .01, *SD* = 24.76; *M(re)*_male_controls = −2.53, *SD* = 28.41; *t*(109) = .50, *p* = .66, n.s.).

We were further interested in observing whether there were any associations between age and time estimates among the patients or the healthy control subjects. Using the median patient age to divide patients into older and younger participant groups, we determined that time estimates were not associated with age in psychiatric patients (*M(age)*_patients = 41 years; *M(re)_age*<41years_patients = 13.02%, *SD* = 53.98; *M(re)_age*>41years_patients = 27.66%, *SD* = 67.29; *t*(101) = 1.21, *p* = .23, *n.s.*).

However, older healthy control participants (*M(age)*_controls = 31 years) produced time interval estimates that were significantly longer than those of younger control participants (*M(re)*_age<31_controls = −8.55, SD = 21.47; *M(re)*_age>31_controls = 5.80, SD = 28.90; t(109) = 2.96, p = .004).

Next, we tested whether the groups of patients with different primary ICD-10 “F” diagnoses differed in their estimates of time. We found that most groups (except for the F1 and F4 groups) overestimated the elapsed time. Overestimation was greatest in the patients with dementia (*M(re)*_F0_patients = 104%, SD = 124.46), whereas the patients with neurotic, stress-related, and somatoform disorders produced rather accurate estimates (*M(re)_F4* = −2.05%, *SD* = 39.71). Due to the small sample sizes, the F0, F1, F6, and F9 subgroups were not included in the ANOVAs. The ANOVAs performed using the primary diagnoses of F2, F3, F4, and F5 as the independent variable yielded no significant differences between the different groups of patients (*F* (3, 94) = 2.02, *p* = .12, *n.s.*).

T-tests comparing the largest psychiatric subgroup of patients with major depression with the healthy subjects revealed significant differences between depressed patients and healthy control subjects (*M(re)_F3_*patients = 17.32, *SD* = 40.61; *M*(re)_controls = −1.18, *SD* = 26.44; *t*(71.96) = 3.00, *p* = .004). Further analysis revealed that these differences can be attributed to gender differences as depressed females and healthy female control subjects showed significant differences (*M*(re)_F3_female_patients = 30.90, *SD* = 40.99; *M*(re)_female_controls = .01, *SD* = 24.76; *t*(47.16) = 3.99, *p* = .000), whereas the same analysis for males yielded no significant results (*M(re)*_male_patients = −8.34, *SD* = 24.90; *M(re)*_male_controls = −2.53, *SD* = 28.41; *t*(68) = −.77, *p* = .44, *n.s.*).

### Descriptive statistics for patients with somatic symptoms

Because there were no significant differences in time estimation performance between the diagnosis groups, we were curious about whether time experience is altered among inpatients in general. Thus, we chose to assess a limited group of patients in non-psychiatric wards who had received no psychotropic medications (for details, see the methods section). Twenty-eight patients (16 females) were asked to take part in the study; 6 patients (4 females) did not participate in the task. The mean age of the participants was 51.3 years (*SD* = 17.1). Patients with somatic symptoms produced rather high estimates on average (21.4%; *SD* 58.25%), and their estimates were significantly different from the estimates made by the healthy control subjects (*t*(131) = −2.92, *p* = .004) but not from the psychiatric patients' estimates (*t*(124) = −.11, *p* = .91, *n.s.*).

### Subjective time experience

Is the measured impairment in time estimation mirrored by changes in the subjective experiences of the assessed subjects? When offered a three-item scale concerning their subjective time experience, the healthy subjects predominantly reported a “normal” time experience. Most psychiatric patients and patients with somatic symptoms reported a subjectively distorted time experience (mostly a decelerated time experience; see table 5). Chi-square tests showed that these group differences in subjective time experiences were highly significant (*Chi^2^* (2, *N* = 79) = 25.93, *p* = .000).

**Table 5:** Subjective general time experience; *Chi^2^* (2, *N* = 79) = 25.93, *p* = .000.

group	normal	decelerated	accelerated	“do not know”
psychiatric patients	5	43	10	7
healthy control subjects	11	3	3	1
control patients with somatic symptoms	10	11	1	-

## Discussion

Touchscreen tablets offer great potential for use in clinical research and can be used to collect meaningful data on time estimation tasks. Several features of touchscreen devices, such as the ability to touch the display with one or more fingers, the ability to capture gestures or motor activities, and the intuitive operation of the device, may facilitate studies with infants, elderly people, or subjects with limited cognitive abilities, thereby opening new areas for clinical studies. However, more methodological research on the practical applications of handheld touchscreen devices is needed. For this reason, the present study was conducted to investigate data collection with touchscreen tablets in a psychiatric setting with a simple time estimation task design.

### Practicality and attendance

One advantage of the use of handheld devices as measuring instruments is their usability for subjects and experimenters. The intuitive operation and the appealing appearance of the iPad may have been advantageous in the present study, especially because refusals to participate were fairly rare (20% of psychiatric patients and 9% of healthy controls). According to the study by Freudenmann and Spitzer 2001 [25] this result can be seen as a high participation rate in a group of psychiatric patients. Psychiatric patients who refused to participate were significantly older than the patients who participated. Elderly individuals hand are still likely to be unfamiliar with computers ([26], even though this study was conducted ten years later) and may have a more skeptical attitude toward electronic devices. Therefore, they may not want to participate in studies carried out with a handheld device. As the usage of mobile devices becomes widespread in the general population [2] and the younger generation is more familiar with computers, one could speculate that age-related refusal to participate in studies with touchscreen tablets will decrease in the coming years. Both the patients and the control subjects often remarked that they appreciated participating in a task with a touchscreen tablet. Even severely depressed patients participated in the task and showed interest and pleasure.

For the examiners, creating and varying parameters for an experimental design is facilitated by the ability to program the device, making it easy to conduct many different tests. This ease of use also creates the risk of programming too many parameters and hampering the clarity of an experimental design; in addition, programming software may be rather expensive.

Another advantage of handheld tablets is their portability, which makes it possible to collect data in various environments in a short period of time. Moreover, their portability enables researchers to take measurements in settings that are familiar to patients and to collect data from patients who are not able to go to a laboratory setting. In psychiatric clinics, patients suffering from mental disorders that require them to stay in a secure ward can participate in studies using the iPad.

### Methodical findings

Randomly generating time intervals every day enabled us to screen timespans for further research. We learned that very short intervals (1–5 s) are prone to overestimation both by psychiatric patients and control subjects; therefore, we excluded these intervals from further analyses. For time intervals between 1 and 5 s, no differences between the psychiatric patients and the healthy control subjects were observed.

However, the sample size in this interval group as well as in the other subgroups, was small. The findings of this study are in line with other findings [18], who showed that patients with affective disorders and healthy control subjects both overestimated time intervals when reproducing a very short interval (1 s), and a study that revealed no differences between groups in the estimation of a 10-s interval [19].

Furthermore, in the present study, long intervals (56–60 s) were estimated fairly accurately by all participants. We attribute this result to the “hint” given in our standardized briefing because the subjects were asked to estimate the number of seconds. In summary, we conclude that very short timespans seem unsuitable for distinguishing psychiatric patients from healthy control subjects, whereas intervals ranging from 15–60 s seem to be more suitable for differentiating between psychiatric patients and healthy individuals. These findings should be considered in future studies.

Randomly generating different time intervals every day avoids bias by preventing the patients from exchanging information about the measurements. When a set combination of time intervals is used, as in previous studies [8] it is likely that information will pass from one inpatient to another. Choosing different intervals every day avoids the possibility of patients exchanging this critical information about the test. Depending on the percentage of patients in a ward within a certain timespan, this procedure may be important.

We show that repetitive measurements can be performed quickly with the method. However, we found a learning effect among the subjects that in some cases could contribute to differences between groups and has in the case of averaging subsequent trials to be considered. In our study repetitive measurements did not compromise the general findings as exclusion of the first data point of the three estimates of each patient did not interfere with overall statistics.

### Duration estimation

Our findings related to time estimation confirm the results of previous investigations that have demonstrated distorted time estimation performances in psychiatric inpatients.

Psychiatric patients continuously overestimated time intervals, whereas healthy control subjects produced fairly accurate estimates. One reason for these differences in average error is the wide range of time estimates (and thus the large standard deviation) produced by the psychiatric patients. The differences between the psychiatric patients and the healthy controls align with other results [17], with the exception that in the present study, no differences in the time estimates between the diagnostic groups could be found. This result may be attributed to a degree of inaccuracy in the diagnoses; to keep the experimental design simple and to focus on the methodological analysis of the iPad, the diagnoses were not peer-reviewed. This is an important limitation of our study.

The influence of characteristics such as age and gender on time sense is particularly interesting. Studies have hypothesized that age has an effect on the perception of time because of the widely held impression that time accelerates with aging, but most studies have reported no significant association between age and time perception [9]. However, our results partially confirm other findings [18], [27] that “age did not have a significant impact” (i.e., time estimation in patients is not associated with age).

In contrast, we found age-related differences in the control subjects; specifically, the younger subjects underestimated timespans, and the older subjects overestimated timespans. This result aligns with results previously obtained with visual stimuli in various healthy control groups [27], [28] and the notion that ageing is associated with time overestimation in time estimation tasks [29].

Several possible explanations for these results could be taken into consideration. The large standard deviation in the psychiatric patient group may obscure possible age effects. Because there were large differences in the subjective time experiences, with most patients reporting a decelerated time experience, this subjective deceleration may overlap with the effects of age on time estimation, even though no direct link between subjective time experience and time estimation error was observed in the present study.

The magnitude and direction of the gender differences in time perception have also been repeatedly investigated clinically and experimentally (see [30]) In the present study, we observed a tendency (although not significant) for the female psychiatric patients to make greater time estimation errors than the male inpatients, who showed distorted but more accurate results. This gender-related difference was not observed in the healthy control group. In other verbal time estimation tasks, results show clearer differences between females and males, with women overestimating timespans [9], [31]. In a detailed summary of previous research concerning this matter, Hancock & Rausch (2010) discuss two explanations. First, an exogenous view of time estimation as a learned ability suggests that the role of women in society has changed markedly in the past few decades. These changes may be associated with changes in time estimation, but no empirical support for this statement exists to date [9]. Second, a more biological explanation is that the “internal clock” works slightly faster in males than in females and thus is associated with differences in time estimation. Because there were no significant differences between our groups, the issue is not solved by our study.

Interestingly, we found an inpatient effect on time estimation. Even though the sample of patients with somatic symptoms was relatively small, the results showing a highly significant difference compared with the healthy control patients are reliable because the statistical method we used takes sample size differences into consideration. We hypothesize that time estimation is severely affected by being an inpatient and that inpatients with somatic symptoms are a suitable control group for psychiatric patients.

### Strengths and limitations of the study

Our study was designed to introduce the iPad as a measurement device for time estimation tasks. A general sample of patients was chosen without using exclusion criteria based on diagnosis. Thus, a strength of the study is that the results depict the typical situation in the hospital we investigated. However, patients often had more than one diagnosis and were treated with multiple medication. Thus, we refrain from making any conclusions in this study regarding the specificity of the impairments in time estimation to the included psychiatric diseases. This limitation of the study is important, especially because the diagnoses were not peer-reviewed. Most of the patients were taking psychotropic medications. It cannot be ruled out that the effects of medication interfered with the patients' accuracy in the time estimation task. The number of psychiatric patients who were not taking psychotropic drugs was too small to draw reliable conclusions. Distorted time estimation in drug-free psychiatric patients has also been found in medication-naive subjects in other studies [32], [33]. Accordingly, we found that patients with somatic symptoms who were not taking psychotropic medication also overestimated times in the task. Of course, duration estimates may also be affected by non-psychotropic medications, and we cannot rule out a systematic effect of other medications (e.g., analgesics) in the small group of non-psychiatric inpatients. In general, our study provides a methodological framework for further studies with defined patient populations.

In summary, three main results emerged from the present study. First, the use of an iPad is an acceptable method for conducting time estimation tasks in psychiatric settings and may offer great potential for further data collection. Second, the duration of the time intervals and the number of trials can influence time estimates. Third, time estimation is significantly distorted in psychiatric inpatients.

The application has been submitted to the appstore and should after acceptance been found by using the search terms “time estimation” and “Preuschoff”. This information will be updated to provide a direct link.
